# Flexor Tendon Rupture Secondary to Gout

**DOI:** 10.1055/s-0043-1772756

**Published:** 2023-10-05

**Authors:** Jeremy V. Lynn, Amy L. Strong, Kevin C. Chung

**Affiliations:** 1 Section of Plastic Surgery, Department of Surgery, University of Michigan Medical School, Ann Arbor, Michigan

**Keywords:** gout, uric acid, tendon insufficiency, flexor tendon rupture, hand

## Abstract

Extra-articular deposition of monosodium urate crystals is a widely recognized manifestation of gout. However, gouty infiltration of flexor tendons in the hand resulting in tendon rupture is exceedingly rare. This case report highlights a patient with gouty infiltration of flexor tendons in the right middle finger resulting in rupture of both the flexor digitorum profundus and flexor digitorum superficialis. Given the extent of gouty infiltration and need for pulley reconstruction, the patient was treated with two-stage flexor tendon reconstruction. Febuxostat was prescribed preoperatively to limit further deposition of monosodium urate crystals and continued postoperatively to maximize the potential for long-lasting results. Prednisone was prescribed between the first- and second-stage operations to prevent a gout flare while the silicone rod was in place. In summary, tendon rupture secondary to gouty infiltration is the most likely diagnosis in patients with a history of gout presenting with tendon insufficiency.

## Introduction

Gout is a crystalline arthropathy characterized by synovial deposition of monosodium urate crystals in the upper or lower extremity. Classically, patients present with acute onset monoarticular joint pain, swelling, and erythema in the absence of inciting trauma. Less common presentations of gout in the hand include flexion contracture, flexor tenosynovitis, and flexor tendon rupture. 1 2 3 4 5 6 While gouty infiltration of flexor tendons is relatively common among patients with uncontrolled disease, there exists only one published report of flexor tendon rupture in the hand secondary to gout. 6 This previously reported case describes a patient with gouty infiltration of the right index finger resulting in rupture of the flexor digitorum profundus (FDP) and flexor digitorum superficialis (FDS). 6 The present case report highlights a patient with gouty infiltration of flexor tendons in the right middle finger resulting in rupture of both the FDP and FDS requiring two-stage tendon reconstruction.

## Case

**Fig. 1:**
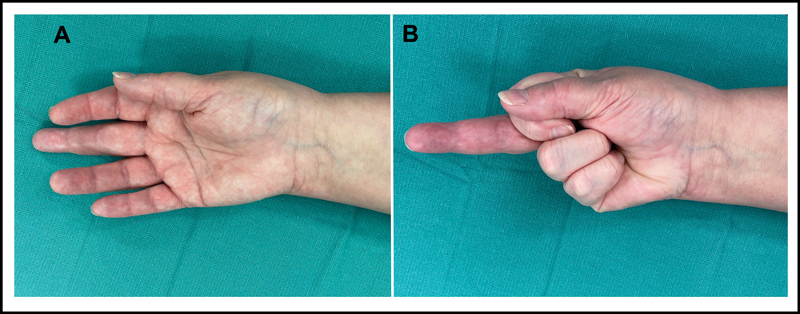
Preoperative physical examination demonstrated generalized swelling of the right middle finger ( **A** ) with inability to actively flex the proximal interphalangeal (PIP) or distal interphalangeal (DIP) joints ( **B** ).

A 57-year-old right hand dominant female with a 2-year history of gout afflicting the right hand presented with 6 months of restricted right middle finger flexion. Her symptoms started following an episode of acute right hand pain and swelling several weeks prior. She denied any history of hand trauma or surgery. Physical examination revealed generalized swelling of the right middle finger with 0 degrees of active flexion of the proximal interphalangeal (PIP) or distal interphalangeal joint ( Fig. 1A, B ). The serum uric acid was 11.1 mg/dL. Radiographic studies demonstrated no abnormalities of the right middle finger. The leading diagnosis was rupture of the FDP and FDS tendons. The patient was encouraged to continue taking febuxostat (80 mg daily) and scheduled for surgery.

**Fig. 2:**
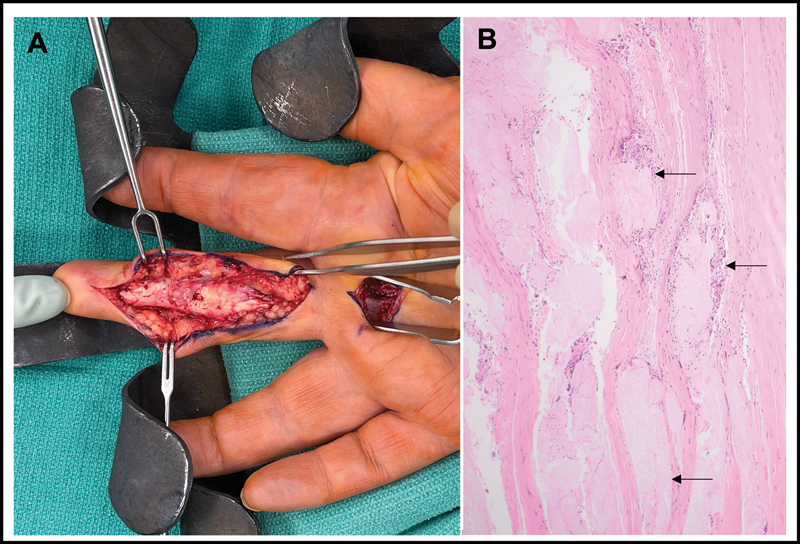
Intraoperative evaluation during the first-stage operation demonstrated extensive gouty infiltration with tendon rupture of both the flexor digitorum profundus and flexor digitorum superficialis ( **A** ). Pathologic analysis demonstrated eosinophilic amorphous gout deposits surrounded by chronic inflammatory cells. The three arrows highlight examples of such deposits on a representative pathology slide ( **B** ).

Following extensive consultation regarding the complexity of injury and demands of postoperative rehabilitation, informed consent was obtained and the patient was taken to the operating room for right middle finger exploration and single-stage versus two-stage flexor tendon reconstruction. Zigzag incisions were designed to expose the involved tendons. Extensive scar tissue was visualized surrounding the pulley system and tendon sheath. The flexor tendon sheath was entered to reveal gross gouty infiltration of the FDP and FDS tendons with zone II rupture ( Fig. 2A ). Both tendons were excised from the lumbrical muscle belly to the distal phalanx. Given the extent of gouty infiltration and need for pulley reconstruction, a two-stage flexor tendon reconstruction was indicated. A silicone rod was passed under the preserved pulleys and sutured to the distal FDP tendon stump. The A2 pulley was reconstructed over the silicone rod with a remnant of excised FDS tendon. The silicone rod was cut proximal to the A1 pulley and left free in the palm to avoid distal rupture. Final pathology confirmed the diagnosis of tophaceous gout ( Fig. 2B ). All cultures were negative. Aggressive passive range of motion exercises were initiated starting 1 week postoperatively. The patient was prescribed prednisone (5 mg daily) in addition to febuxostat (80 mg daily) between the first- and second-stage operations.

**Fig. 3:**
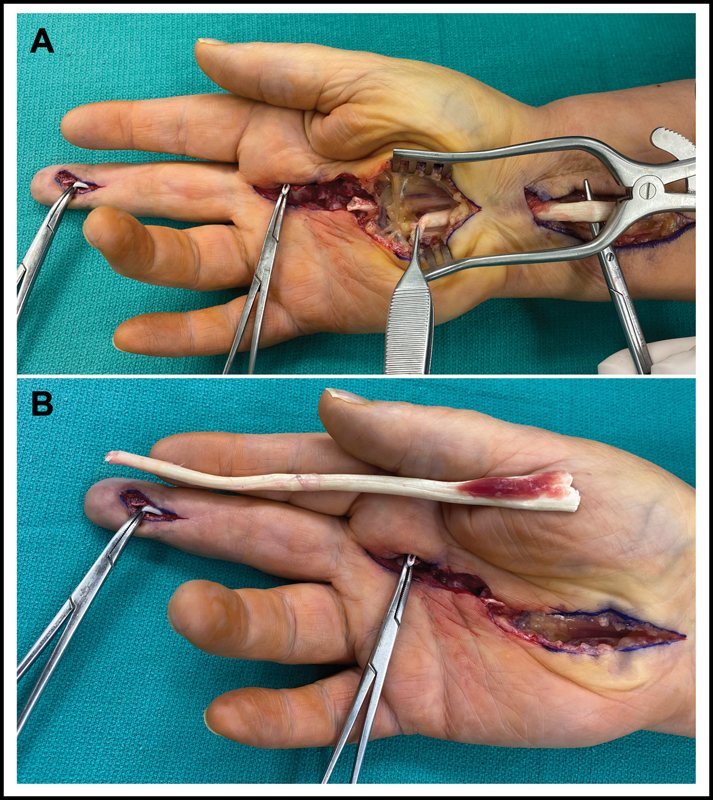
Intraoperative photographs during the second-stage operation demonstrating the flexor digitorum superficialis tendon graft in situ ( **A** ) and along the planned site of inset ( **B** ).

Approximately 3 months later, the patient returned to the operating room for silicone rod removal and tendon grafting. The previous incisions were opened to reveal the silicone rod gliding smoothly within a well-established pseudosheath. The palmaris longus tendon was absent in the right wrist. Therefore, the remaining proximal FDS tendon was selected for use as a tendon graft. The carpal tunnel was released to ensure the proximal FDS tendon remnant was safely identified and dissected free prior to division of the FDS tendon at the myotendinous junction ( Fig. 3A, B ). The tendon graft was split longitudinally to decrease the tendon bulk and passed from proximal to distal through the pseudosheath. The distal end of the tendon graft was secured to the distal phalanx using a tie-over button bolster. Specifically, a Prolene suture was placed in the tendon using the Bunnell technique, and the suture ends were passed through the distal phalanx using two Keith needles. Then, the suture ends were tied over a sterile button on the nail plate at maximal tension to secure the tendon end to the bone. The proximal end of the tendon graft was secured to the distal aspect of the native FDP tendon in the palm using a Pulvertaft weave technique. Tension was set with the middle finger in slightly greater flexion compared with the normal cascade. The patient was then placed in a dorsal blocking splint for 6 weeks to protect the reconstruction. Hand therapy following the Duran protocol was started 1 week postoperatively. Febuxostat (80 mg daily) was continued postoperatively.

The patient was examined in clinic 1 month postoperatively and found to have limited active flexion of the PIP joint (∼45 degrees) with a passively correctable swan neck deformity of the right middle finger. Additionally, a small draining wound was identified on the volar aspect of the distal phalanx. The patient was prescribed a 10-day course of amoxicillin-clavulanic acid and instructed to continue aggressive hand therapy to improve her range of motion. She was subsequently evaluated in clinic 4 months postoperatively. Physical examination of the right middle finger demonstrated complete resolution of the draining wound, yet persistence of the limited active flexion of the PIP joint (30 degrees) and associated swan neck deformity. The tendon reconstruction appeared to be intact, suggesting the A2 pulley reconstruction may have failed resulting in decreased active flexion of the digit. This scenario highlights the importance of aggressive postoperative hand therapy to achieve full range of motion. The patient was counseled on the risks and benefits of repeat pulley reconstruction, and she expressed her desire to delay further operations.

## Discussion

The flexor tendon sheath has been theorized to serve as a barrier against gouty infiltration of flexor tendons. 7 However, recent publications have demonstrated that gouty infiltration can penetrate the flexor tendon sheath leading to flexion contracture, flexor tenosynovitis, and flexor tendon rupture. 1 2 3 4 5 6 The first and only previously reported case of flexor tendon rupture secondary to gout was presented by Wurapa and Zelouf in 2002. 6 The authors performed a single-stage flexor tendon reconstruction given the intact flexor tendon sheath and the patient's complex past medical history. 6 In the present case report, we further demonstrate that gouty infiltration of the flexor tendon sheath may result in disruption of the underlying flexor tendon with eventual tendon rupture. The extent of gouty infiltration and need for pulley reconstruction in our patient required two-stage flexor tendon reconstruction.

Preoperatively, tendon rupture secondary to gouty infiltration should be high on the differential diagnosis among patients with suspected or confirmed gout presenting with tendon insufficiency. In patients lacking a formal diagnosis of gout, the presence of needle-shaped negatively birefringent monosodium urate crystals on joint aspiration is confirmatory. It is essential to optimize the medical treatment of gout preoperatively to minimize further deposition of monosodium urate crystals. Baseline control of gout is achieved with a xanthine oxidase inhibitor such as febuxostat or allopurinol. Additionally, patients should be encouraged to avoid purine-rich foods (i.e., red meat and alcohol). Gout flares are treated with a combination of nonsteroidal anti-inflammatory medications (i.e., indomethacin), colchicine, and glucocorticoids. Many of the medications used to treat gout flares have side effects that must be considered. For example, salicylates may inadvertently decrease uric acid excretion and colchicine may cause gastrointestinal or neuromyopathic symptoms. Importantly, xanthine oxidase inhibitors should not be started during an acute gout flare to avoid transiently worsening symptoms. Our patient presented with a known history of gout and her rheumatologist had previously started her on febuxostat (80 mg daily). Therefore, we reinforced the importance of strict compliance with febuxostat at the initial consultation given our concern that gout was contributing to the patient's tendon insufficiency.

Intraoperatively, it is important to thoroughly evaluate the flexor tendon for gouty infiltration and associated adhesions given the variable involvement of the pulley system and flexor tendon sheath. Gouty infiltrates should be debulked to the extent necessary to facilitate smooth motion of the involved tendon. Extensive adhesions between the flexor tendon and surrounding flexor tendon sheath may require tenolysis or tendon excision with reconstruction to restore range of motion, even in the absence of gross tendon rupture. 8 In the case of flexor tendon rupture, single-stage versus two-stage flexor tendon reconstruction is determined based on the extent of gouty infiltration of the pulley system and tendon sheath. Regarding the present case report, we performed a two-stage flexor tendon reconstruction given the severely damaged flexor tendon bed and need for pulley reconstruction. 9 With respect to tendon grafting, Wurapa and Zelouf placed a short tendon graft from the less involved proximal FDP stump to the less involved distal FDS stump. 7 During our stage two flexor tendon reconstruction operation, we placed a lengthy segment of proximal FDS tendon from the distal phalanx to the proximal end of the native FDP tendon in the palm as the patient lacked a palmaris longus tendon. It is important to recognize that variable flexor tendon involvement in the affected digit results in unpredictable tendon graft requirements.

Postoperatively, excised segments of flexor tendon and flexor tendon sheath should be sent to pathology for analysis. The specimens must be fixed in alcohol rather than formalin, as urate crystals are destroyed by routine formalin processing. Histologic analysis of specimens excised during our first-stage operation demonstrated eosinophilic amorphous gout deposits surrounded by chronic inflammatory cells. The presence of chronic inflammatory cells on histologic analysis highlights the mechanism of tendon rupture in patients with gout. Specifically, monosodium urate crystals within the flexor tendon sheath and underlying flexor tendon induce a local inflammatory response. Excessive inflammation causes macrophages to remain in the proinflammatory (rather than profibrotic) state, and excessive macrophage-mediated remodeling eventually weakens the tendon resulting in rupture. 10 The infiltration of cells from the intrasynovial tendon sheath to the injured tendon during the healing process accounts for the dense adhesions we encountered during the first-stage operation. 10

In summary, the present case report highlights a patient with gouty infiltration of flexor tendons in the right middle finger resulting in rupture of both the FDP and FDS. This article provides additional evidence that the flexor tendon sheath does not preclude flexor tendons from gouty infiltration. Thus, when patients with a history of gout present with tendon insufficiency, tendon rupture secondary to gouty infiltration is the most likely diagnosis.
